# Case report on successful ‘bail out’ aortic homograft implantation in a 81-year old woman with aortic ring rupture after double TAVI procedure

**DOI:** 10.1186/s13019-015-0233-x

**Published:** 2015-03-04

**Authors:** Piotr Kołsut, Andrzej Juraszek, Piotr Brzozowski, Maciej Dąbrowski, Adam Witkowski, Jacek Różański, Mariusz Kuśmierczyk

**Affiliations:** 1 Department of Cardiac Surgery and Transplantation, The Cardinal Stefan Wyszyński Institute of Cardiology, Warsaw, Poland; 2 Department of Interventional Cardiology and Angiology, The Cardinal Stefan Wyszyński Institute of Cardiology, Warsaw, Poland

**Keywords:** TAVI, Aortic ring rupture, Aortic homograft, Bail out aortic surgery

## Abstract

A 81-year woman was admitted to our institution due to worsening chronic heart failure. The patient presented herself with severe aortic valve stenosis and mitral valve insufficiency. Due to estimated high operative risk a TAVI approach was chosen. Following the fist TAVI implantation (Sapien XT™ 26 mm) a big paravalvular leakage was diagnosed. Hence, in order to close the paravalular leakage, a second TAVI procedure with a Core Valve™ 26 mm was performed. A following CT scan showed signs of aortic ring rupture. We therefore decided to perform open heart surgery. After removal of both TAVI prosthesis, native valve excision a 21 mm sized aortic homograft was implanted. Additionally, a mitral valve annuloplasty with CE Physio™ 28 mm ring was performed. In the postoperative period the patient remained for three weeks in the ICU, followed by two weeks on general ward. The patient was discharged home in good condition, with good left ventricular function and regular homograft function in the aortic position. The open heart surgery should have had been performed primarily. Nevertheless, the decision on the treatment strategy is always difficult in case of borderline patients.

## Background

Transcatheter aortic valve implantation (TAVI) represents an accepted alternative method of intervention in patients with aortic valve disease and high operative risk [1]. Some studies also showed the promise of performing TAVI on lower-risk patients [2]. The cardiovascular and all-cause mortality is reported to be similar to conventional surgery at early and long-term follow-up [3]. Nevertheless, TAVI is still associated with many complications. Aortic annulus rupture is considered to be the most severe form and reported in approximately 2% cases [4]. The only treatment option available for this complication is surgical replacement of the aortic root. We present a case of an 81-year-old woman with aortic annulus rupture and a false aneurysm after double unsuccessful TAVI procedure. This is a rare case of good clinical outcome after delayed surgical bail-out treatment.

## Case presentation

An 81 year-old female with severe aortic stenosis and moderate mitral insufficiency, persistent atrial fibrillation, peripheral varicosis, bad clinical state and general fragility was reviewed by the local heart team. Conventional open heart surgery was considered to be a high risk approach by the multidisciplinary team. The calculated Euro Score II value was 6.45%. The aortic annulus diameter was 22 mm and the left ventricular ejection fraction was good. Due to the patient’s poor clinical state a TAVI was applied.

**Figure 1:**
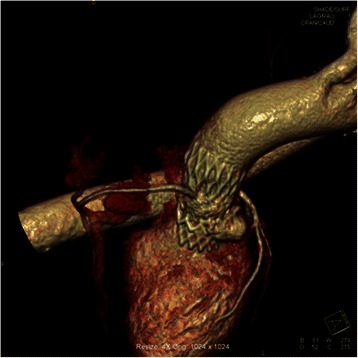
**CT scan after the 2nd TAVI.** A pseudoaneurysm is located in the area of the left sinus Valsalvae.

After ballon-pre-dilatation, a 26 mm Edwards Sapien XT™ valve was implanted. The postoperative TTE showed a significant paravalvular leakage in the posterior part of the aortic annulus. The left ventricular function was preserved and there was no pericardial effusion. The patient’s hemodynamical situation remained stable. After a short intensive care unit (ICU) stay signs of advanced heart insufficiency occurred. In order to close the paravalvular leakage a second TAVI was performed using a 26 mm CoreValve™. TEE after valve implantation showed a decline of the paravalvular leakage. Subsequently the patient was transferred to the ICU. In the following days the patient’s hemodynamical condition worsened and administration of adrenaline and dopamine was required. Nonetheless the patient showed NYHA IV symptoms, and the CT-scan showed an additional 14×13×12 mm pseudoaneurysm located in the area of the left sinus Valsalvae (Figure 1). After re-evaluation of the patient an open heart approach was chosen.

**Figure 2:**
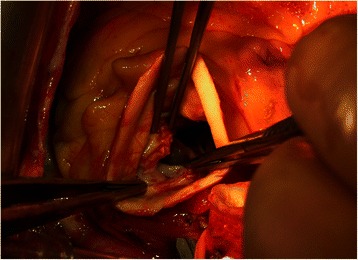
**Inspection of the aortic root showing the valve annulus rupture.**

The procedure was performed via median sternotomy access with normothermic extracorporeal circulation (ECC) and bicaval cannulation. A standard aortotomy was performed. During aortic cross clamp time cold blood cardioplegia was administered directly to the coronary ostia. Both TAVI prostheses were removed and the native valve was excised. The inspection showed a rupture of the aortic annulus with a pseudoaneurysm formation (Figure 2). The rupture was located in the area of the left-right coronary commisure. The pseudoaneurysm was found in the periannular area and continued along the posterior part of the annulus under the ostium of the left coronary artery. Due to the advanced anatomical situation an extra-anatomic homograft implantation was favored. A 21 mm sized aortic homograft was implanted using the root-replacement technique with single-pledged Prolene™ 4.0 sutures. The homograft placement excluded the area of the annular rupture and the entry to the pseudoaneurysm. Additionally, mitral valve annuloplasty with CE Physio™ 28 mm ring was performed due to enhanced mitral regurgitation. The patient was weaned successfully from the ECC. The ECC time was 239 minutes and the aortic cross clamp time 208 minutes. Afterwards the patient was transferred to the ICU under small dobutamine support.

The recovery time in the ICU lasted three weeks, followed by two weeks on general ward. As the patient had moderate renal insufficiency preoperatively, in the postoperative period temporary increase of the renal dysfunction was observed. The patient developed a postcardiotomy syndrome requiring longer time of pericardial drainage and pharmacotherapy with colchicine and steroids. TEE assessment showed good left ventricular fraction, regular homograft function and a good result of mitral valve reconstruction. The patient was referred home in good condition.

### Discussion

Despite the patient was referred to the TAVI procedure due to her bad clinical state and general fragility it should be emphasized that a patient with an Euro Score II value of 6.45% should primarily be considered for a standard surgical aortic valve replacement. Additionally, an untreated mitral valve insufficiency may have a negative impact on mid-term results after TAVI procedure itself.

Albeit some reports discuss conservative treatment options for patients with aortic annulus ruptures after TAVI, these strategies seem only to work out in fully stable patients [5]. In our case-report we present a patient, with a worsening hemodynamical situation despite best pharmacological support. We decided to perform open heart surgery on our patient, as it occurred to be the only curative option, despite the high mortality rate of 45% described in the literature [6].

Furthermore this case underlines the importance of an early angio-CT scan performance, as it fast helped to find the right diagnosis of the annulus rupture. This observation is also well documented in literature [5]. The second TAVI was applied as TEE examination underestimated the aortic situation by only identifying a paravalvular leakage.

The annular diameter was small, 22 mm. The calcium was mostly located on the annulus and in the commisures. At our hospital the 26 mm Sapien XT™ valve was the smallest one. We suppose that the prosthesis diameter was too large and it caused the annular rupture during the baloon dilatation. Performing the second TAVI after the first unsuccessful one was of no benefit for the patient. Definitively, the rescue operation should have been performed directly after the first TAVI procedure. Probably, the essential lesson of this case is the necessity to perform an broad imaging work-up followed by an early rescue surgery in such patients. Here, the use of aortic homograft is a feasible option to replace a destroyed aortic root. An alternate option to the abovementioned is the use of a stentless aortic root bio-prosthesis.

As the mitral insufficiency was enhanced by the ventricular dilatation we decided to perform the mitral ring implantation as no remodeling was expected and the procedure does not take too much time in experienced hands.

## Conclusions

The patient described above should have had open heart surgery primarily. Nevertheless, the decision on the treatment strategy is always difficult in case of borderline patients.

Rupture of the aortic ring after TAVI is a rare and potentially lethal complication. Patients with progressive clinical signs of hemodynamical deterioration of paravalvular leakage after TAVI shall undergo a broad imaging work-up. In case of a valve ring rupture, the rescue surgery should be performed immediately after the primary TAVI failure. Aortic homograft implantation shall be considered, as it provides an adequate tool to replace the destroyed aortic root.

## Consent

Written informed consent was obtained from the patient for the publication of this report and any accompanying images.

## Abbreviations

CT: Computed tomography
TAVI: Transcatheter aortic valve implantation
ECC: Extracorporeal circulation
ICU: Intensive care unit
TTE: Transthoracic echocardiogram
NYHA: New York Heart Association
